# Towards Complete Sets of Farnesylated and Geranylgeranylated Proteins

**DOI:** 10.1371/journal.pcbi.0030066

**Published:** 2007-04-06

**Authors:** Sebastian Maurer-Stroh, Manfred Koranda, Wolfgang Benetka, Georg Schneider, Fernanda L Sirota, Frank Eisenhaber

**Affiliations:** 1 Research Institute of Molecular Pathology, Vienna, Austria; 2 VIB, SWITCH Lab, Flanders Interuniversity Institute for Biotechnology, Brussels, Belgium; 3 Vrije Universiteit Brussel, Brussels, Belgium; 4 ULB, Service de Conformation des Macromolécules Biologiques et de BioInformatique, Brussels, Belgium; Columbia University, United States of America

## Abstract

Three different prenyltransferases attach isoprenyl anchors to C-terminal motifs in substrate proteins. These lipid anchors serve for membrane attachment or protein–protein interactions in many pathways. Although well-tolerated selective prenyltransferase inhibitors are clinically available, their mode of action remains unclear since the known substrate sets of the various prenyltransferases are incomplete. The Prenylation Prediction Suite (PrePS) has been applied for large-scale predictions of prenylated proteins. To prioritize targets for experimental verification, we rank the predictions by their functional importance estimated by evolutionary conservation of the prenylation motifs within protein families. The ranked lists of predictions are accessible as PRENbase (http://mendel.imp.univie.ac.at/sat/PrePS/PRENbase) and can be queried for verification status, type of modifying enzymes (anchor type), and taxonomic distribution. Our results highlight a large group of plant metal-binding chaperones as well as several newly predicted proteins involved in ubiquitin-mediated protein degradation, enriching the known functional repertoire of prenylated proteins. Furthermore, we identify two possibly prenylated proteins in Mimivirus. The section HumanPRENbase provides complete lists of predicted prenylated human proteins—for example, the list of farnesyltransferase targets that cannot become substrates of geranylgeranyltransferase 1 and, therefore, are especially affected by farnesyltransferase inhibitors (FTIs) used in cancer and anti-parasite therapy. We report direct experimental evidence verifying the prediction of the human proteins Prickle1, Prickle2, the BRO1 domain–containing FLJ32421 (termed BROFTI), and Rab28 (short isoform) as exclusive farnesyltransferase targets. We introduce PRENbase, a database of large-scale predictions of protein prenylation substrates ranked by evolutionary conservation of the motif. Experimental evidence is presented for the selective farnesylation of targets with an evolutionary conserved modification site.

## Author Summary

Various cellular functions require reversible membrane localization of proteins. This is often facilitated by attaching lipids to the respective proteins, thus anchoring them to the membrane. For example, addition of prenyl lipid anchors (prenylation) is directed by a motif in the protein sequence that can be predicted using a recently developed method. We describe the prediction of protein prenylation in all currently known proteins. The annotated results are available as an online database: PRENbase. A ranking of the predictions is introduced, assuming that existence of a prenylation sequence motif in related proteins from different species (evolutionary conservation) relates to functional importance of the lipid anchor. We present experimental evidence for high-ranked human proteins predicted to be affected by anticancer drugs inhibiting prenylation.

## Introduction

Protein prenylation is facilitated by three eukaryotic enzymes with partially overlapping substrate specificities [1–3]. Farnesyltransferase (FT) and geranylgeranyltransferase I (GGT1) recognize the so-called C-terminal CaaX box of substrate proteins to attach either a farnesyl (15 carbons) or geranylgeranyl (20 carbons) anchor to the conserved cysteine via a thioether linkage. Rab geranylgeranyltransferase or geranylgeranyltransferase II (GGT2) requires the formation of a complex of the substrate protein with a dedicated escort protein, REP (Rab escort protein) [4], and typically attaches two geranylgeranyl anchors to C-terminal cysteines in motifs such as -XXXCC, -XXCXC, -XXCCX, -XCCXX, or -CCXXX [5]. Isoprenyl lipid anchor attachment to C-termini of proteins not only serves for membrane targeting but can also be crucial for protein–protein interactions [6]. Inhibition of protein prenylation is a promising approach for developing anti-cancer drugs [7] as well as for treating parasitic diseases [8,9]. Therefore, it is of great scientific and applied medical interest to clarify which proteins and pathways are affected by farnesyl- or geranylgeranyltransferase inhibitors in human cells or in unicellular parasites.

Based on the refinement of descriptions of sequence motifs recognized by the three enzymes (FT, GGT1, and GGT2) in substrate proteins, we have recently developed amino acid sequence–based predictors for various types of protein prenylation (PrePS [10]). PrePS is available as a WWW service (http://mendel.imp.ac.at/sat/PrePS/index2.html). Since the rate of false-positive predictions of PrePS is low (for proteins with CXXX C-terminus, the false-positive rate is estimated at ∼5% at a sensitivity for true targets of ∼98% [10]), this tool is appropriate for large-scale automated annotation (for example, for proteome scans). In this work, we apply PrePS to finding all potential protein substrates of the three prenyltransferases. With the analyses of these protein sets, it can be determined which prenylation targets are preferentially affected if enzyme-specific prenyltransferase inhibitors are applied.

As previous experience with a similar project (the application of the MyrPS/NMT myristoylation predictor [11,12] for searching the nonredundant database and the resulting MYRbase [13]) has shown, large-scale scans produce a considerable number of hits, and, for their ranking with respect to the biological significance, additional criteria are necessary. It should be noted that the score function of PrePS tests the concordance of C-termini of query proteins (the terminal 12 residues) with a simplified binding site model of the respective prenyltransferase without consideration of other sequence properties. It is not rare that sites for posttranslational modifications and sequence motifs coding for subcellular translocation are not conserved among proteins with otherwise highly similar sequences (exemplary cases of myristoylation [13], GPI lipid anchoring [14], and prenylation [15]). More surprisingly, functional motifs can be hidden in proteins without the proper biological context and be masked by other sequence signals (e.g., the case of peroxisomal targeting signal type 1 (PTS1) in proteins destined for other subcellular localizations [16]). Nevertheless, conservation of the prenylation site among a larger number of homologues will indicate enhanced biological importance of the potential lipid modification and increase the confidence in correct prediction (evOluation concept in MYRbase [13]). Therefore, evolutionary conservation of prenylation sites in homologous families can be used for ranking in hit lists and for the selection of potential targets for experimental verification in conditions of limited resources.

Here, we report the results obtained after applying the three prenylation predictors over the National Center for Biotechnology Institute's (NCBI) nonredundant protein sequence database (NR). The proteins predicted to be prenylated have been clustered into homologous families and are made available as the annotated database PRENbase. A sophisticated interface can generate target lists with regard to the experimental status of the modification (known, predicted, etc.), exclusive or shared types of modifying enzymes (FT, GGT1, GGT2), as well as for evolutionary conservation by constraining the taxonomic distribution within clusters or for single sequences. We investigate the validity of various hit-ranking schemes relying on sequence homology information and taxonomic distribution. Finally, we use PRENbase to list human proteins that could represent elusive cellular targets of FT inhibitors (lack of alternative prenylation by GGT1 under FT inhibition) [17] and verify experimentally the prenylation status of selected human proteins (versions of Rab28, the BRO1/rhophilin domain containing FLJ32421 [termed BROFTI], Prickle1, and Prickle2) following our published protocols [18].

### PRENbase: Methodological Workflow and Database Description

**Figure 1:**
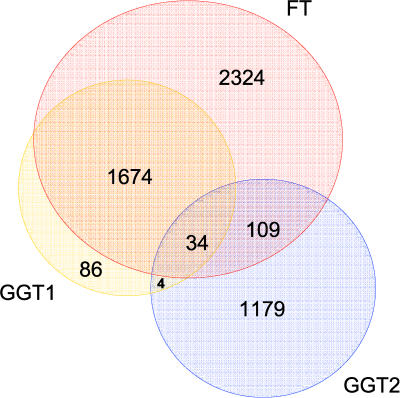
Distribution of Predicted Substrate Proteins among the Three Prenyltransferases

The three predictors included in PrePS [10] have been run over the NR at NCBI. After removing protein fragments with an incomplete C-terminus (as annotated in Genbank), 5,410 proteins were predicted to be prenylated. Figure 1 shows the distribution among the three modifying enzymes, including their substrate protein overlaps. While the number of predicted substrates shared between FT and GGT1 is not surprising (mainly due to the fact that FT can also prenylate substrates with terminal leucine [10]), it is interesting that there also is a substantial overlap with GGT2. At least for Rab8 and Rab11, this enzyme ambiguity has been demonstrated in vitro [19,20]. It has to be mentioned that the predictions by PrePS merely represent the capability of a substrate sequence to be modified when presented to the enzyme. In vivo, activity, relative affinity, and availability of FT, GGT1, and GGT2 in the cellular context determine which enzyme will execute the prenylation for a given substrate protein.

#### Family clustering.

To facilitate the selection of targets for experimental validation, we tried to rank the predictions by the importance of the lipid anchor for their function based on the analysis of evolutionary motif conservation within protein families. It would be of special interest to study the conservation of farnesyl, geranylgeranyl, and double geranylgeranyl anchors within protein families, as this can indicate exclusive or overlapping substrate specificity between the three enzymes. Thus, the extent of variation can give additional hints on the importance of the specific anchor size [8]. We employed BLAST [21] and the MCL (Markov chain clustering) algorithm [22] to assign the 5,410 predicted sequences to a total of 1,024 clusters (protein families). For details on the clustering procedure, see the Materials and Methods section.

#### Annotation of families of predicted homologous prenylation targets.

We have manually curated protein family annotations for clusters with at least three sequences (201 clusters total). Due to the power law–like behavior of protein family cluster sizes [13], we could provide curated cluster annotation for approximately 83% of the predicted sequences by looking at only 20% of all clusters. The remaining clusters of size 1 or 2 have been annotated with names automatically extracted from their description lines.

In addition to the protein family name and function description, we annotated clusters with respect to verification status. This is not a trivial task because it requires manual lookup of hundreds of literature sources. While the actual number of experimentally verified proteins is small compared with the total number of predictions, many proteins can safely be assumed to be prenylated simply by similarity to known examples. We annotate clusters/families as KNOWN (+) when they include at least one from a list of 113 proteins experimentally verified to be prenylated. In addition, we created the annotation category LIKELY (*) for clusters that do not have an experimentally verified example included directly, but where members of the cluster show a clear similarity (BLAST E-value < 1e−10) to at least one of the verified cases. Finally, clusters without any detectable similarity to any of the 113 proteins experimentally verified to be prenylated are categorized in PRENbase as NEW (?). While the former families (with annotation KNOWN and LIKELY) form a basis to summarize existing knowledge of prenylated proteins, the latter (NEW) are of special interest because their function apparently has not been recognized yet in the context of prenylation.

During the annotation process, we have also encountered a few predictions where conservation of a C-terminal cysteine in CaaX box arrangement can also occur for prenylation-independent functions such as disulfide bridges (e.g., metridin-like ShK toxin family members). Although these do not appear to be prenylation targets in vivo, it cannot be excluded that they become prenylated in a different context when their C-termini would be exposed to the prenylating enzyme. The endothelin-converting enzyme 1 (ECE1) from the neprilysin-like zinc metallopeptidase family is another example with a CaaX box where the capacity for prenylation is apparently not used in vivo (possibly because of a disulfide bond). It is predicted by PrePS to be weakly prenylated and, indeed, its C-terminus has been shown to be weakly prenylatable in vitro [15]. However, it is known to be a type II transmembrane protein. Therefore, the C-terminus and, hence, the potential prenylation motif moves to the lumenal side of the endoplasmic reticulum membrane and becomes inaccessible for the prenyltransferases. In agreement with the cellular context, the protein does not appear to be prenylated in vivo [15,23]. Thus, these predictions are not necessarily false positives. We have annotated these predictions in PRENbase as OUT-OF-CONTEXT (−).

#### Family ranking.

If a predicted protein feature, such as a prenylated C-terminus, is conserved among a large number of homologues (large cluster size), this feature appears more critical for biological function and more reliably predicted. Thus, predictions can be scored by

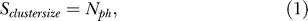
where *N_ph_* is the number of family members with a predicted prenylation site. However, the number of homologous proteins sharing the motif becomes less indicative for ranking purposes when the protein family in general is overrepresented in nature or in the databases, respectively (e.g., immunoglobulin chains). Hence, knowledge of the total family size, including proteins with and without the investigated motif, can be used to balance for such overrepresentation. As suggested earlier [24], ranking of families by evolutionary motif conservation could be performed with a scoring function such as

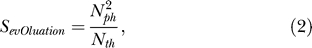
where *N_ph_* is the number of predicted and *N_th_* is the total number of homologues or family members. This ratio balances for overrepresented sequences when compared with the simple ranking by cluster size (Equation 1). The square of *N_ph_* also helps to downrank very small clusters or orphans relative to clusters with large *N_ph_*; e.g., *N_ph_* as well as *N_th_* is 1 in these cases.

Instead of ranking based on counting the number of homologues, it is also possible to analyze the taxonomic distribution and score the families according to how widespread (or old) the motif is in the evolution of the protein family. Such phylogenic complexity can simply be estimated as a score function of the number of species (*N_spec_*) that have family members with the motif. To remove artificial bias introduced through disproportional sequencing coverage of specific proteins of closely related species, we suggest multiplying *N_spec_* by a factor that evaluates the broad distribution throughout all kingdoms and selected divisions. In our case, we count how many of 12 selected taxonomic groups from all kingdoms (archaea, bacteria, viruses, mammals, birds, amphibiae, fishes, insects, nematodes, fungi, plants, and “other eukaryotes”) are covered by the investigated family (*N*_12_ ≤ 12). Then, the final phylogenic complexity scoring function can be written as:

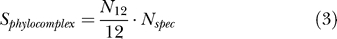



It should be noted that ranking based on phylogenic complexity does not require the computationally costly determination of the total family size (including members without the motif). Large clusters that consist mainly of sequences of closely related species are downranked in favor of families with a more widespread taxonomic distribution.

**Figure 2:**
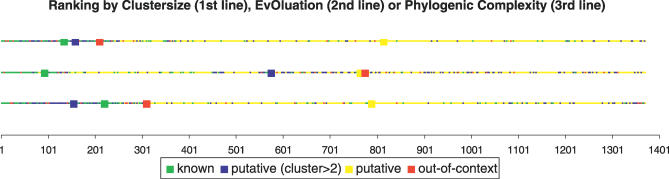
Performance of Different Ranking Schemes for Clusters with Predicted Prenylation Targets

To investigate the performance of the different ranking schemes, we plotted the distribution of clusters colored by their annotated modification status (Figure 2). Clusters that are homologous to proteins that have already been shown experimentally to be prenylated are shown in green. Those without known prenylated homologues are colored blue (or yellow if the cluster size is smaller than three). Clusters where the motif appears conserved for prenylation-independent functions are colored red. The median values for the distribution of the different cluster and ranking types are marked in Figure 2 and listed in Table 1.

**Table 1:**
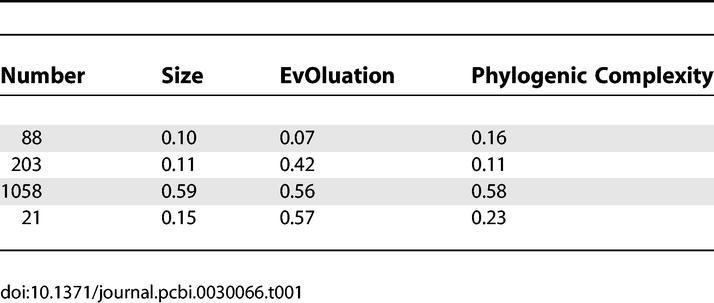
Values of Cluster Medians from Figure 2

It can be seen that the simple ranking by cluster size brings the known or likely prenylated proteins (green clusters) to the front of the list. However, the red clusters also appear to be highly ranked. Using the evOluation score [13] for ranking retains the green in front and moves the red to the back. The phylogenic complexity approach performs somewhat worse in downranking the red clusters, but, in contrast, it keeps larger unknown clusters (blue) closer to the top of the list. In conclusion, the different ranking schemes substantially influence the distribution of clusters and might be used to select targets based on emphasis of specificity of the motif for the complete protein family (evOluation [13]) or on taxonomic diversity (phylogenic complexity).

#### Estimate of gain of performance when adding evOluation to PrePS.

We previously estimated that PrePS misses about 2% of yet unknown prenylation motifs (cross-validated average sensitivity of PrePS: 98%) while predicting only 0.1% false positives in complete database searches (average specificity of PrePS: 99.9%) [10]. This estimate for false positive predictions includes motifs that can be prenylated in vitro, while the in vivo context makes the lipid modification rather unlikely. In this work, we identified and discussed such examples (see the previous subsection Annotation of families of predicted homologous prenylation targets). We referred to these predictions as OUT-OF-CONTEXT rather than as false positives. Since there are no new cutoffs and the “evOluation” score is only used for a priority ranking of all predictions, the absolute rates of false negative and false positive predictions in PRENbase are by definition the same as those reported for the PrePS method. We do, however, show that the evOluation score widens the gap between true positives (KNOWN) and contextual false positives OUT-OF-CONTEXT in the ranking. This is visualized in Figure 2, quantified in Table 1, and further discussed in the previous paragraphs.

**Figure 3:**
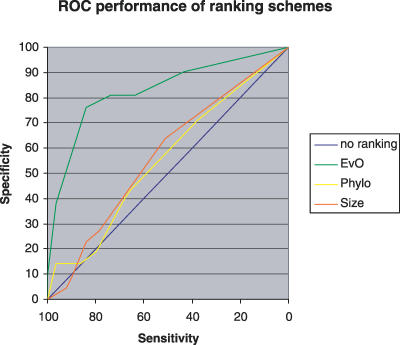
Performance Gain of Ranking Schemes over Standard PrePS without Ranking

To estimate the performance gain of adding the evOluation ranking compared with the standard PrePS prediction alone, we apply ROC analysis by sliding an artificial threshold over the cluster ranks and count the true positive (KNOWN) and contextual false positive (OUT-OF-CONTEXT) clusters above or below the given thresholds. This allows plotting sensitivity (100-rate of false negatives) versus specificity (100-rate of false positives) for the different methods (Figure 3). As can be seen, the evOluation score clearly outperforms the other ranking schemes and results in a gain in contextual specificity of up to 60% at high sensitivities compared with the standard PrePS. Apparently, random occurrences of small motifs, a typical source of false positives, are indicated by a lower conservation within their protein family, and this feature can therefore be used to further reduce false positives in the context of the biological importance of the motif for the protein. We propose that similarly significant performance gains could also be reached for other methods predicting small sequence motifs by considering the evolutionary conservation of the predicted motifs within protein families.

#### PRENbase Web interface.

**Figure 4:**
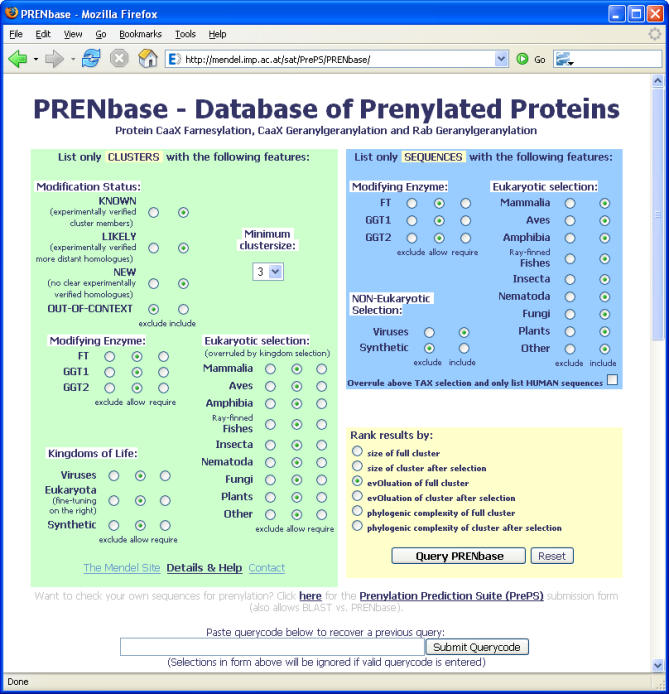
Screenshot of PRENbase Query Interface

The manually annotated clusters/families of prenylated proteins described above are available as PRENbase. A Web interface (Figure 4, http://mendel.imp.univie.ac.at/sat/PrePS/PRENbase/) has been designed to allow sophisticated queries to PRENbase: (1) for the experimental status of the modification (KNOWN/LIKELY/NEW/OUT-OF-CONTEXT); (2) for the range of prenyltransferasesexclusive or shared types of modifying enzymes (FT, GGT1, GGT2); as well as (3) for evolutionary conservation by constraining the taxonomic distribution within clusters or for single sequences. The output can be ranked by cluster size, by the evOluation score that also takes into account the total family size in databases, or by an estimated phylogenic complexity. The default settings give access to the collection of both known and predicted eukaryotic and viral prenylated proteins, which can then be browsed. To facilitate tasks for less-experienced users, we have listed a series of standard queries that might be of particular biological interest. Queries are assigned to a unique query code that can be used to recover previous queries without having to readjust the multiple parameters of the interface. Furthermore, users can map their own sequence against PRENbase using a BLAST module linked to the PrePS server (http://mendel.imp.univie.ac.at/sat/PrePS/).

#### HumanPRENbase.

**Figure 5:**
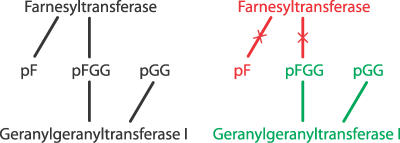
Altered Prenylation under FT Inhibition

For biomedical applications, it is of great interest to know which human proteins are particularly affected by prenyltransferase inhibitors that have already passed phase II and III clinical trials [25] but whose molecular mode of action is not fully understood yet [2]. For example, farnesyltransferase (FT) inhibitors can abolish the prenyl modification only for substrates that cannot be alternatively modified by GGT1 (Figure 5). The classical examples are (1) H-Ras that can only be modified by FT (hereafter, proteins of this type are called pF) and (2) K-Ras that can be a substrate of both FT and GGT1 (hereafter, pFGG). The distinction of pF- and pFGG-type proteins is critical since it helps to identify the exclusive cellular targets affected by FT inhibition (pFs) and give hints to the molecular mechanisms involved in various cancer types [2,17].

The most prominent group of prenylated oncogenes comprises members of the Ras superfamily of small GTPases. In PRENbase, these are clustered together in a small number of large families with high *S_evOluation_* (Equation 2) and *S_phylocomplex_* (Equation 3). This example shows that it is useful to clearly identify the orthologous counterparts of individual human proteins. A procedure to derive clusters of such orthologous groups is described in detail in the Materials and Methods section and has allowed the creation of a list of 242 unique human clusters with their isoforms and in-paralogues merged together in the same cluster with at least one (human) member predicted to be prenylated. We removed sequences that are less than 50% of the length of the query sequence (in cases of multidomain proteins) to avoid ambiguous cluster assignments of short homologous sequences. Furthermore, the resulting clusters made up by the orthologues and a representative human sequence are available in a style similar to the original PRENbase. The listed status annotation is derived using the same criteria as for the general PRENbase clusters. For example, there are few members of the large Ras, Rab, and Rho families of GTPases where the prenylation has been shown directly (annotated as KNOWN). However, for many other related clusters (annotated as LIKELY), prenylation can often be safely inferred if a valid motif exists. On the other hand, clusters annotated as NEW signify that this protein family is not yet known to be prenylated and could involve a completely new mode of action for prenyltransferase inhibitors. This HumanPRENbase (http://mendel.imp.univie.ac.at/sat/PrePS/HumanPRENbase/) can now be queried for the experimental status of the modification of homologues (known/new…), and exclusive or shared types of modifying enzymes (FT, GGT1, GGT2) as well as for evolutionary conservation by constraining the taxonomic distribution within clusters or for single sequences. The output can also be ranked by cluster size, by evOluation, score or by phylogenic complexity.

## Results/Discussion

### Review of Previous Knowledge of Prenylated Proteins

In total, we have collected a list of at least 113 individual proteins experimentally verified to be prenylated that are part of 41 “KNOWN” clusters, and similarity to these justifies the annotation as “LIKELY” for another 106 clusters in PRENbase. Thus, a major strength of this work is the complete proteomic view of prenylation with an added evolutionary perspective. For example, by querying PRENbase for families with conserved prenylation motif in mammals, insects, nematodes, fungi, and plants, we derive a core set of only three clusters of already known prenylated proteins. These are the Rab, the Rho/Rac, and the DnaJ-like heat shock chaperone families which, therefore, could be postulated as being the oldest examples of prenylated proteins due to their most widespread taxonomic distribution. When weakening the conservation requirements and “only” considering conservation in mammals, insects, and nematodes, several other families join this list of presumably important prenylated proteins. These are (in the order of the evOluation ranking): the Ras/Ral/Rap family, the Lamin B cluster (linking also more generally coiled coil proteins), a cluster of mixed serine/threonine kinases, geranylgeranylated G gamma subunits, protein tyrosine phosphatase IVA, protein phosphatase 1 regulatory subunit 16 (in cluster with other Ankyrin domain containing proteins), as well as phosphorylase B kinase α+β subunits. Although spread over multiple clusters due to their sequence diversity, fungal mating factors/pheromones compose another large functionally related group of prenylated proteins.

In contrast to the examples above where the prenylation site is highly conserved among various taxa, there are many cases where the predicted prenylation is specific to taxonomic lineages or even single species. Nevertheless, this posttranslational modification can be an important requirement for function of the respective proteins. Therefore, the smaller clusters that can be found in PRENbase also merit deeper investigation.

### “The Mother of Ras”

It is no surprise that the small GTPase families, well-known for their prenylation, top the evolutionary ranked lists in PRENbase. Apparently, multiple duplication events of common prenylated ancestor genes led to the numerous paralogous proteins in the Ras superfamily of small GTPases, resulting in the observed phylogeny of function [26,27].

Although the historical research focus [26] is clearly on the Ras subfamily due to the oncogenic potential of its most famous members H-Ras and K-Ras, the evolutionary history paints a different picture of importance of the Ras/Rap, Rho/Rac, and Rab families. The Rab family [27] is not only the most populated one in PRENbase (followed by the Rho/Rac subfamily), but it also has a much wider taxonomic distribution. In fact, there are no Ras proteins in plants, while there are several different Rabs and some Rac homologues spread in the plant kingdom [28]. Although highly speculative and by no means unambiguously conclusive, one can attempt to narrow down the candidates for closest living relatives of the common ancestor of Ras GTPases by searching for the taxonomically most conserved individual Ras-related proteins. In HumanPRENbase, the respective hits are (in decreasing order of the phylogeny-based ranking): Rab1B, Rab7, Rac1, and Rab6A. So the “mother of Ras” would have been more likely to be related to Rab or Rac proteins nowadays. Since Rab proteins are typically dually geranylgeranylated by the type II prenyltransferase GGT2 and both Ras and Rac proteins are specifically processed by type I prenyltransferases FT and GGT1, the similarity of substrate characteristics would point to a closer relationship of Rac to Ras, rather than to Rab proteins. In agreement with the co-clustering of Ras and Rac proteins in phylogenetic tree analyses, including other Rab and more distantly related GTPases, Ras proteins appear to have emerged from a common ancestor shared with the Rho/Rac family.

### The Anonymous “Known” Group of Plant Copper Chaperones

In our predictions for prenylated protein families, we find a large group of 88 homologous plant proteins that are annotated to be metal-binding copper chaperones spread over 21 clusters. Surprisingly, the mainstream prenylation-related publications have not mentioned these proteins as prenylated, so far. A thorough search of the literature, however, reveals that a previous work has already shown prenylation for three of these proteins (all in soybean) [29]. Therefore, the corresponding clusters of related proteins appear in PRENbase annotated as “KNOWN” or “LIKELY,” respectively. Functional characterization of this protein family appears scarce, and given the large number of members and the additional information of a conserved prenylation motif, their likely importance should be subject to further investigations.

### Predictions with New Functional Context for Prenylation

Our approach identifies 979 sequences in 114 clusters that do not share similarity with already known prenylated proteins and whose predicted prenylation, therefore, would expand the possible functional repertoire of prenylated proteins in cells. Surprisingly, we find several proteins that are related to ubiquitin-mediated protein degradation.

One of these groups comprises some ubiquitin-like proteins. In particular, UBL3 and its prenylation motif are not only conserved in organisms from mammals to insects and worms but, apparently, also in some fungi and plants. Fitting into the related functional context of ubiquitin-mediated degradation, it is also interesting to observe predicted prenylation for several ubiquitin hydrolases. For example, ubiquitin specific protease 32 is conserved in mammals, pufferfish, and insects with a domain architecture of an N-terminal EF-hand domain, a central DUF1055 domain, followed by a C-terminal ubiquitin hydrolase domain which finally precedes the conserved prenylation motif. Furthermore, we predict several fungal proteins that have a carboxy-terminal ubiquitin hydrolase domain in addition to a prenylation motif. Interestingly, there also exists an E2 ubiquitin-conjugating enzyme with conserved prenylation motif in *Arabidopsis* and rice.

The connection of prenylation and protein degradation continues with the prediction of a prenylation site in F-box and leucine-rich repeat proteins, with FBL2 being conserved in organisms from mammals to insects, worms, and fungi. These proteins typically serve as adaptors targeting substrate proteins of SCF (skip-cullin-F-box) and analogous degradation complexes [30].

Besides proteins with already known functions, a conserved prenylation motif is also valuable information for proteins with domains of unknown functions. Most prominently in our list, proteins containing a DUF544 domain appear conserved in organisms from mammals to worms, plants, and fungi. In another cluster, integral membrane proteins from mammals, insects, and worms share a DUF1339 domain together with the prenylation motif.

### Selection and Experimental Verification of Human FTI Targets

The selection of candidates for experimental verification focuses on predictions related to possible human target proteins for FTIs, because of the implications for important upcoming cancer therapeutics [25]. Figure 5 depicts the different types of prenylation substrates distinguished by their enzyme preference, which determines the effectiveness of FT inhibition. While H-Ras has long been seen as a primary target for FTIs, it has become clear that other proteins are affected as well, and the hunt is on for these elusive FTI targets [17].

**Figure 6:**
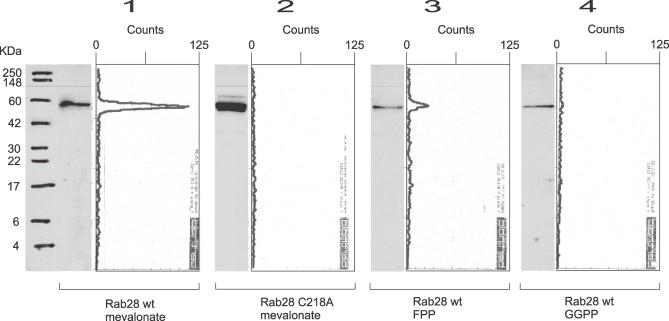
Western Blots and TLC Scanning Results for Rab28 with Radioactive Prenyl Anchor Precursors Western blot and corresponding scans from TLC linear analyzer of wild-type GST-Rab28-fusion protein translated with [^3^H]mevalonic acid (lane 1), GST-Rab28 C218A with [^3^H]mevalonic acid (lane 2), GST-Rab28 with [^3^H]FPP (lane 3), and GST-Rab28 with [^3^H]GGPP (lane 4). There is significant incorporation of a product of mevalonic acid as well as FPP, while incorporation of GGPP is not detectable, suggesting that Rab28 is primarily a farnesylation target.

**Figure 7:**
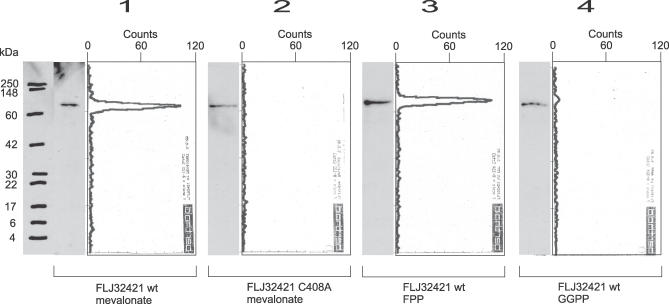
Western Blots and TLC Scanning Results for FLJ32421 (BROFTI) with Radioactive Prenyl Anchor Precursors Western blot and corresponding scans from TLC linear analyzer of wild-type GST-FLJ32421-fusion protein translated with [^3^H]mevalonic acid (lane 1), GST-FLJ32421 C408A with [^3^H]mevalonic acid (lane 2), GST-FLJ32421 with [^3^H]FPP (lane 3) and GST-FLJ32421 with [^3^H]GGPP (lane 4). There is significant incorporation of a product of mevalonic acid as well as FPP, while incorporation of GGPP is close to the detection limit, suggesting that FLJ32421 (BROFTI) is primarily a farnesylation target.

**Figure 8:**
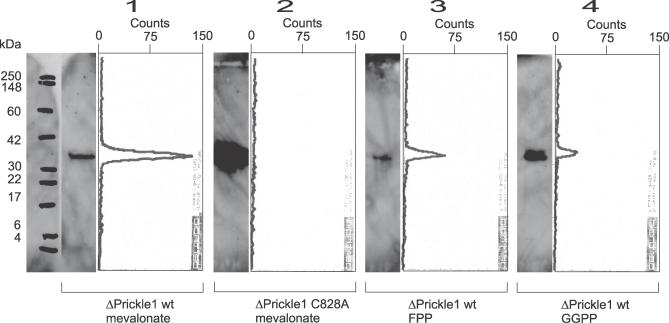
Western Blots and TLC Scanning Results for a 15 Amino Acid C-terminal Fragment of Prickle1 with Radioactive Prenyl Anchor Precursors Western blot and corresponding scans from TLC linear analyzer of wild-type GST-ΔPrickle1 fusion protein translated with [^3^H]mevalonic acid (lane 1), GST-ΔPrickle1 C828A with [^3^H]mevalonic acid (lane 2), GST-ΔPrickle1 with [^3^H]FPP (lane 3) and GST-ΔPrickle1 with [^3^H]GGPP (lane 4). There is significant incorporation of a product of mevalonic acid as well as FPP, while incorporation of GGPP is lower despite a higher total amount of protein in the latter case, suggesting that Prickle1 is primarily a farnesylation target.

**Figure 9:**
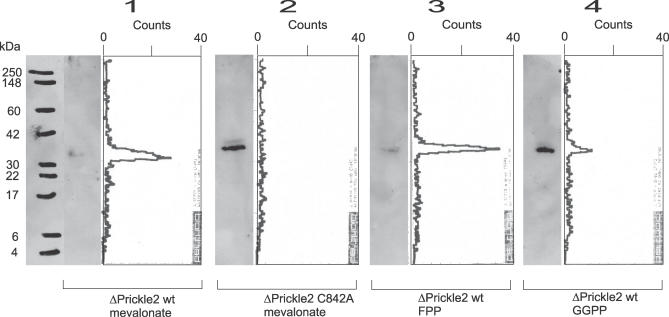
Western Blots and TLC Scanning Results for a 15 Amino Acid C-terminal Fragment of Prickle2 with Radioactive Prenyl Anchor Precursors Western blot and corresponding scans from TLC linear analyzer of wild-type GST-ΔPrickle2-fusion protein translated with [^3^H]mevalonic acid (lane 1), GST-ΔPrickle2 C842A with [^3^H]mevalonic acid (lane 2), GST-ΔPrickle2 with [^3^H]FPP (lane 3), and GST-ΔPrickle2 with [^3^H]GGPP (lane 4). There is significant incorporation of a product of mevalonic acid as well as FPP, while incorporation of GGPP is lower despite a higher total amount of protein, suggesting that Prickle2 is primarily a farnesylation target.

**Figure 10:**
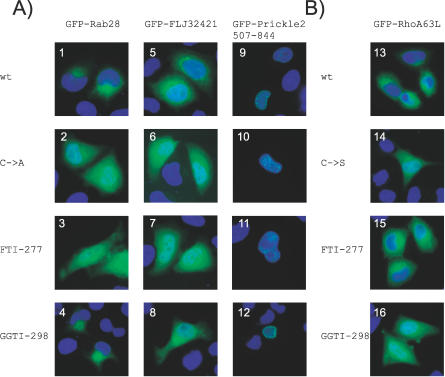
Localization of N-terminal GFP Constructs of Rab28, FLJ32421/BROFTI, Prickle2 (507–844), and RhoA63L in HeLa Cells HeLa cells were analysed by fluorescence microscopy after transfection with the following constructs: inserts 1, 3, and 4—GFP-Rab28; insert 2—GFP-Rab28 C218A; inserts 5, 7, and 8—GFP-FLJ32421; insert 6—GFP-FLJ32421 C408A; inserts 9, 11, and 12—Prickle2; insert 10—GFP-Prickle2 C841A; inserts 13, 15, and 16—GFP-RhoA63L (as positive control for a geranylgeranylated target); insert 14—GFP-RhoA63L C190S. The GFP-RhoA plasmids were kindly provided by Channing J. Der (University of North Carolina Chapel Hill, Chapel Hill, North Carolina, United States). Nuclei were co-stained with DAPI (blue color). (A) GFP-Rab28, GFP-FLJ32421, and GFP-Prickle2 are membrane-localized with (4, 8, 12) or without (1, 5, 9) GGTI-298 treatment. Mutation of the Cys in the CaaX box (2, 6, 10) or treatment with FTI-277 (3, 7, 11) cause mislocalization and accumulation of the fusion proteins in the nucleus. (B) GFP-RhoA is membrane-localized with (15) or without (13) FTI-277 treatment. Mutation of the Cys in the CaaX box (14) or treatment with GGTI-298 (16) cause mislocalization and accumulation of RhoA in the nucleus.

The experimental verification of prenylation predictions follows a new, recently described methodology [18] based on fast scanning of the incorporation of ^3^H-labelled prenyl precursors with a thin layer chromatography (TLC) analyzer. Details are given in the Materials and Methods section. Conceptually, we test the site of prenylation by comparing the incorporation of ^3^H-labelled mevalonate (general prenyl anchor precursor) in the wild-type protein and a mutant protein where the predicted prenylated cysteine is mutated to alanine (Figures 6–9, lanes 1 and 2). The type of prenyl anchor preferentially attached to the target proteins in vitro is tested by comparing the incorporation of ^3^H-labelled farnesylpyrophosphate (farnesyl anchor precursor) and geranylgeranylpyrophosphate (geranylgeranyl anchor precursor), respectively (Figures 6–9, lanes 3 and 4). Furthermore, we investigate the role of prenylation for in vivo localization of GFP-tagged target proteins (Figure 10). Besides the wild-type and cysteine-mutant protein, we also analyze the effect of farnesyltransferase and geranylgeranyltransferase inhibitors on localization of the wild-type protein. From a true FTI target (pF) we expect the same mislocalisation phenotype with cysteine mutation and under FT inhibition, but no phenotype with GGT inhibitor (Figure 10A).

Table 2 shows the predicted human FTI targets (pFs as defined in Figure 5), top-ranked by evOluation score, and with numerical and taxonomy statistics of the cluster of orthologues. Several well-known prenylated proteins are among the top ten on the list. NAP1-like 1 (first) has recently been shown to be farnesylated [31]. H-Ras (second) is the classically known FTI target [7]. Also, prenylation of phosphorylase kinase β (fourth, [32]), Dexras1 (fifth, [33]), DnaJ/Hsp40 homologues (seventh, [34]), and certain transducins (tenth, [35]) is well established.

**Table 2:**
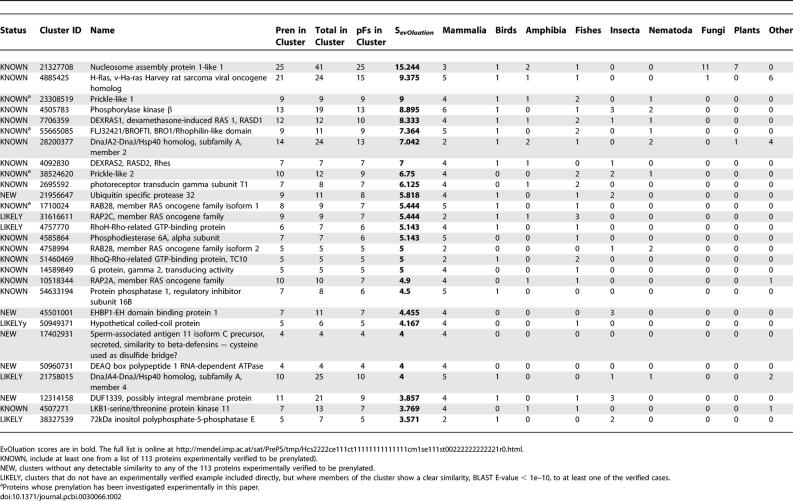
High-Ranked Predicted FTI-Targets (pFs) Sorted by EvOluation Score with Cluster and Taxonomy Statistics

The selective preference of RasD2 (eighth) for farnesyl anchors has been unambiguously shown in our previous work [18]. Direct experimental evidence for the prenylation of Prickle1 (third), the BRO1-domain containing cluster (sixth), Prickle2 (ninth), as well as for another important protein, the Rab28 short isoform (12th), is provided here (see paragraph below). Thus, the experimental verification of the prenylation status of the top clusters is completed with this work.

In humans, Rab28 exists in at least two isoforms, differing in an insertion at the C-terminus. They are distantly related to the Rab proteins (∼30% sequence identity), which are important in vesicle fusion and targeting. While the short isoform is expressed in most tissues, the long isoform is predominately found in testis [36]. As the enzymological tests in vitro show (Figure 6), Rab28 (motif: -CAVQ) can be prenylated exclusively by FT. This conclusion is supported also by in vivo cell culture studies (as well as for Prickle2 and FLJ32421/BROFTI; see Figure 10).

The in vitro experimental study provides direct evidence that FLJ32421 (motif: -CYIS), a hypothetical human protein, is a preferential farnesylation target (Figure 7). The protein contains a BRO1/Rhophilin-like domain that is known to interact with Rho proteins (which often carry prenyl anchors themselves [37]), and the lipid anchors could generally serve to co-localize the binding partners [13]. We suggest the name BROFTI instead of the generic FLJ32421 in tribute to its domain architecture and prenyltransferase substrate characteristics.

Prickle1 (motif: -CIIS, Figure 8) and Prickle2 (motif: -CIIS, Figure 9), the human homologues to the prickle gene of Drosophila melanogaster [38], are both preferential farnesylation targets. In fly, the gene product is important for establishing planar cell polarity [39,40]. Similar functions in cell polarity have been demonstrated in frog (Xenopus laevis) [41], zebrafish (Danio rerio) [42], and ascidians (Cioni savignyi) [43], indicating that the function in human might also be in the localization of the planar cell polarity proteins Frizzled and Dishevelled. The CaaX box in zebrafish Prickle was already shown to be important for localization of the protein [42].

While we have tested the prenylation status of evolu-tionarily widely conserved, high-ranking examples in our list, there are in total 128 human proteins that serve as predicted FTI targets. The full list is available online at (http://mendel.imp.ac.at/sat/PrePS/tmp/Hcs2220ce111ct11111111111111cm1se200st00202222222221r3.html)

### Dual FT/GGT1 Targets Unaffected by FT Inhibition

As opposed to pFs, pFGGs are classified due to their ability to be prenylated by either FT or GGT1 (Figure 5). These pFGGs include (1) proteins with motifs ending in Leucine that are better GGT1 than FT substrates, as well as (2) proteins that are normally farnesylated in the cell (better FT substrates), but can be alternatively prenylated by GGT1 if FT is inhibited. Table 3 lists the top 15 of the latter group. Among these are oncogenic proteins such as K-Ras and N-Ras, with the severe result that FTI inhibitors are ineffective against associated cancers.

**Table 3:**
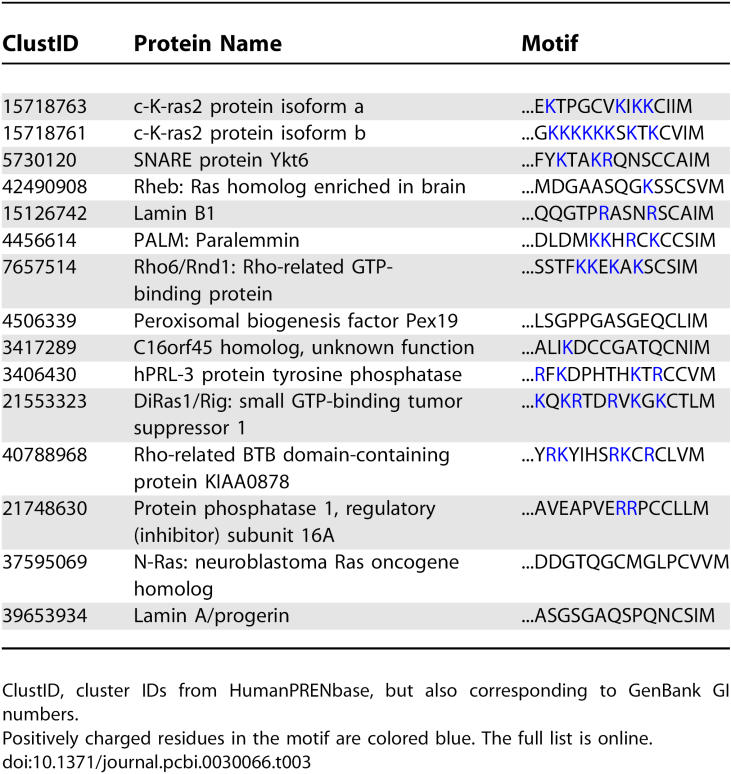
High-Ranked FT Substrates Predicted To Be Unaffected by FT Inhibition

### New Viral Proteins Processed by Eukaryotic Host Enzymes

Previously, the only known examples of prenylation of viral proteins by the eukaryotic host were the Hepatitis Delta large antigen and viral variants of H-Ras and K-Ras, as well as the US2 tegument protein of bovine Herpes viruses.

Surprisingly, our search reveals two candidate proteins from Mimivirus, a giant virus in amoebae that might be a pneumonia-associated human pathogen [44,45]. The first predicted prenylated Mimivirus protein is most closely related to Rab GTPases, while the second is a DnaJ-like molecular chaperone. This particularly large DNA virus is known for its extraordinary gene content normally absent in other viruses [46]. Since there is no similarity to the prenylating enzymes in the Mimivirus genome, the predicted prenylation motifs would only be able to receive a lipid anchor by eukaryotic host enzymes. In light of the fact that several Mimivirus proteins originate from horizontal gene transfer from eukaryotes [47], it is interesting to note that the Rab-like protein is most similar to Rabs found in three different Alveolata species, while the DnaJ-like protein has its closest homologues in Trypanosomes. If, indeed, the prenylation motif would have remained functional and been processed by eukaryotic host enzymes, FTI inhibitors could eventually affect the lipid modification of the DnaJ-like protein whose -CAQQ motif cannot be prenylated by enzymes other than farnesyltransferase.

While there are several other predictions of prenylation motifs in viral proteins (170 sequences in 46 clusters), it is difficult to estimate the likelihood of their functionality, given the requirement that eukaryotic host enzymes be available. Hence, we are more confident in predicted prenylation motifs in proteins that are at least homologous to proteins with known prenylation in Eukaryotes. As an additional example to the above Mimivirus proteins, we find an ankyrin domain–containing protein with FT-specific prenylation motif conserved in canarypox and fowlpox virus.

### Importance of Specific Prenyl Anchor Length and Evolutionary Exchangeability

Farnesyl (C15) and geranylgeranyl (C20) anchors differ in length by one isoprene unit (C5). However, this difference does not seem to matter for some proteins, such as the yeast a-factor mating pheromone [48] and RhoA [49]. On the contrary, importance of the specific prenyl anchor length has been shown, at least, for G gamma 1 and 2 [50], rhodopsin kinase [51], H-Ras [52,53], R-Ras [53], and RhoB [54]. Besides the change in hydrophobicity and altered membrane affinity, the cause of the length dependency might lie in specific interactions with prenyl-binding domains of other proteins [6].

In PRENbase, we observe that protein families differ in the evolutionary exchangeability of farnesyl and geranylgeranyl anchors. While there are several pFGG families where both anchor types are predicted to occur, there are a few pF-only families where farnesyl anchors appear to be the strongly preferred lipid type. From the above list of known examples for length dependency, we find that only G gamma 1 and 2 have a purely conserved farnesyl preference. While for rhodopsin kinase only the chicken orthologue switched to geranylgeranyl, there are several lower eukaryotes with an H-Ras orthologue ending in a geranylgeranylation motif. R-Ras and RhoB end with a -CXXL motif that by itself already can be substrate of either FT or GGT1.

At the same time, the a-factor mating pheromones, where anchor length should be less important, also appear in pF-only families, which, however, could be due to the confinement of clustering together only very closely related species lacking evolutionary time to diverge. The same probably applies to the many almost identical large subunits of Hepatitis delta virus, which are clustered into a pF-only family. On the other hand, the FT restriction also represents a possible vulnerability to FT inhibitors.

Given the above listed ambiguities, one cannot conclude with certainty whether a specific prenyl anchor length is important for a protein family based on the evolutionary variability of substrate preferences. However, in a taxonomically widely conserved family, a clear preference for farnesylation could still indicate a length dependency and, consequently, a requirement of farnesyl for specific protein–protein interactions. In HumanPRENbase, besides the above mentioned G gamma 1 and 2, the following families fall under these criteria: nucleosome assembly protein 1-like 1, prickle-like 1, phosphorylase kinase β, FLJ32421/BROFTI, RasD2/Rhes, RhoH, Rab28 long isoform, RhoQ, EH domain binding protein 1, DnaJ-homolog A4, 72kDa inositol polyphosphate-5-phosphatase E, and WD+tetratricopeptide repeats protein 1.

### Conclusions

PRENbase provides (1) a review of previous knowledge of known and likely prenylated proteins resulting in the rediscovery of the large group of prenylated metal-binding chaperones in plants; (2) target lists for experimental validation of newly predicted prenylation are ranked by evolutionary conservation, which leads to the notion that several proteins involved in ubiquitin-mediated protein degradation could be prenylated; (3) lists of possible targets for FT inhibition (human proteins that are unique substrates of FT and not GGT1 or GGT2) with the experimental evidence for Prickle1, Prickle2, the BRO1-domain-containing FLJ32421 (termed BROFTI), and Rab28 (short isoform); (4) lists of dual FT/GGT substrates that are essentially not affected by FT inhibition or that can receive an altered anchor type under FT inhibition; (5) a list of viral proteins possibly processed by eukaryotic host enzymes, most notably two proteins from Mimivirus; as well as (6) examples of the importance of specific farnesyl anchor length (clusters that only include FT but not GGT1 or GGT2 substrates) that could be indicative of involvement in protein–protein interactions.

## Materials and Methods

### PRENbase family clustering procedure.

In MYRbase, sequences with higher than 40% sequence identity have been clustered into protein families. This rather conservative threshold is reasonable to infer similarity of biological function [55–57] with confidence, but frequently leads to spreading protein families with many remotely similar members and from phylogenetically distant taxa over several clusters. In the case of PRENbase, we applied the MCL procedure to unite many of these clusters [22] and to facilitate a more comprehensive phylogenetic comparison. The use of BLAST [21] allows finding more remote homologues, but simple single linkage clustering based merely on significant BLAST similarity cannot account for the multidomain modular architecture of proteins. For example, frequently occurring regulatory domains (such as SH3) can appear in different contexts with other domains. Therefore, proteins with different functions would be clustered together according to their similarity in such a single overlapping domain hit. These problems seem to have been largely overcome by the MCL algorithm [22] that allows for a certain flexibility of intercluster BLAST connections that are weaker than respective average intracluster links. More precisely, the MCL method understands sequences as nodes in a graph with edges between nodes weighted by the negative logarithm of the BLAST E-value of the two sequences (the average of backward and forward searches); hence, their sequence similarity. The graph is transformed into a matrix with edge weights being normalized to probabilities of walking between nodes. When simulating random walks within the graph, walks within clusters are much more likely than walks between clusters. Through iterative expansion and inflation of the matrix (until the matrix essentially remains unaltered by further iterations), the links within clusters are strengthened and intercluster connections downweighted.

To cluster predicted proteins into their natural families independent of the existence/prediction of a lipid anchor, we have executed BLAST searches (E-value 0.005) starting with the 5,410 predicted proteins against the same complete database from which the predictions were derived (NCBI's NR with 2,179,151 entries, based on GenBank/GenPept version 144). Using the measured BLAST similarity as input for MCL [22], with the inflation parameter I set to 5.0 (for fine-grained clusterings, best precision in a benchmark of clustering SCOP families [22]), we obtain 1,024 clusters. For comparison, single linkage clustering of the 5410 proteins at an E-value threshold of 0.005 would result in 615 clusters, merging several clusters compared with the MCL clustering.

### HumanPRENbase orthologue clustering.

We first generated a list of human proteins that are predicted to be prenylated by at least one of the three enzymes FT, GGT1, and GGT2 by running PrePS over NCBI's NR. Then, we determined the orthologues in other organisms with the condition of best reciprocal BLAST hits. The algorithm employed here follows in the steps of earlier methods to detect orthology and paralogy relationships [58,59], employing the definition of orthologues and in-paralogues as in [60]. This scheme, however, is not straightforward due to several problems. First, the reciprocal similarity search started with the nonhuman organism might find an isoform or in-paralogue (duplication has occurred after the last speciation event) of the initial human query as best hit. We have found that a threshold of greater than or equal to 98% identity (within the aligned segments) is a reasonable threshold with which to classify isoforms. In-paralogues were identified as human proteins that occur as BLAST hits with an E-value smaller than E-50 (this threshold is set to limit the number of noninformative reciprocal BLASTs) and that find the initial query or find one of its known isoforms in a reciprocal BLAST before proteins of any nonhuman species. Finally, we define orthologues as proteins that are the best hits of their species to a human query protein and that in a back-BLAST find either the initial query or its isoforms or in-paralogues as best human hit.

### Construct production and cloning.

We generated plasmids containing GST and pEGFP fusions of all genes studied in this work. The cDNAs of Rab28 short isoform and FLJ32421/BROFTI were cloned into the pGEX5X1-vector, thereby creating N-terminal GST-fusion proteins. Since the cDNAs received for Prickle1 and Prickle2 did not match or only partially matched the desired sequence, we used oligonucleotides representing the last 15 residues at the C-terminus instead. The Stratagene QuikChange XL Site-Directed Mutagenesis Kit was used to introduce a cysteine-to-alanine mutation in the CaaX motif. Since this residue is the site of covalent thioether linkage of the isoprenoid modification, the ability to become modified should be abolished. Both wild-type and mutant cDNA of Rab28 short isoform and FLJ32421/BROFTI were also cloned into the pEGFP C2 vector. For Prickle2, we used a C-terminal fragment representing the last 338 residues at the C-terminus, which is the longest matching sequence we had available. The N-terminal GFP-fusion proteins were used to investigate the subcellular localization in transiently transfected HeLa cells. No GFP-construct of Prickle1 was cloned, since the localization of the last 15 amino acids would not have been representative at all.

### In vitro prenylation assay.

The cDNA of the GST fusion proteins was amplified by PCR and transcribed and translated in vitro using the Promega TNT Quick Coupled Transcription/Translation Kit in the presence of the radioactive label of choice (typically, 20 μCi [^3^H]mevalonic acid, 10 μCi [^3^H]FPP, or [^3^H]GGPP, all purchased from American Radiolabeled Chemicals, http://www.arc-inc.com). The target protein was purified using glutathione sepharose 4B-beads (75% slurry, from Amersham Biosciences, http://www.gelifesciences.com), precipitated with ice-cold acetone and resuspended in sample buffer. After SDS-PAGE and transfer to a nitrocellulose membrane by electroblotting, the incorporated label was detected using a Berthold TLC linear analyzer LB 282. The protein yield was detected by standard Western blotting techniques (primary antibody: anti-GST-antibody from rabbit, 1:5,000; secondary antibody: ECL Anti-rabbit IgG, Horseradish peroxidase linked whole antibody from donkey purchased from Amersham Biosciences, 1:10.000; ECL plus Western Blotting Detection Kit solution, Hyperfilm ECL from Amersham Biosciences).

### Determination of intracellular localization.

HeLa cells were transfected with the GFP-expression vector constructs for Rab28 short isoform, FLJ32421/BROFTI and Prickle2 using Lipofectamine and Plus Reagent in serum-free medium (Life Technologies, http://www.invitrogen.com). The cells were grown to sufficient density, fixed, permeabilized, washed, and mounted in vectashield (Vector Laboratories, http://www.vectorlabs.com). Localization of the fusion proteins was investigated by fluorescence microscopy. The effect of farnesylation and geranylgeranylation inhibitors was assessed by treatment of the cells with FTI-277 (10 μM) or GGTI-298 (5 μM) (Sigma, http://www.sigmaaldrich.com). All experimental procedures were performed as previously described [18].

## Supporting Information

### Accession Numbers

Accession numbers (IMAGE clone ID) of cDNA clones from the RZPD clone libraries (http://www.rzpd.de/products/clones) used in this paper are: FLJ32421/BROFTI (IMAGp961F02139Q2), Prickle1 (IMAGp998C0210744Q1), Prickle2 (IMAGp686N1787Q2), and Rab28 short isoform (IMAGp686F1021Q2).

Accession numbers from GenBank (http://www.ncbi.nlm.nih.gov/Genbank) of mRNA corresponding to the cDNA clones used in this paper are: Rab28 short isoform (NM_001017979), Prickle1 (NM_153026), Prickle2 (NM_198859), and FLJ32421/BROFTI (NM_144695).

Accession numbers of clusters from PRENbase (http://mendel.imp.ac.at/sat/PrePS/PRENbase) mentioned in this paper are: a-factor mating pheromones (6324184), mammalian, insect and nematode UBL3s (22137475), fungal and plant UBL3s (50912815), ubiquitin specific protease 32 (13560797), fungal ubiquitin hydrolases (50426781) and (40744684), E2 ubiquitin-conjugating enzyme from *Arabidopsis* and rice (50918253), F-box and leucine-rich repeat proteins (47218849), proteins containing a DUF544 domain (35193062 and 40740499), integral membrane proteins from mammals, insects, and worms sharing a DUF1339 domain (20521916).

Accession numbers of clusters from HumanPRENbase (http://mendel.imp.ac.at/sat/PrePS/HumanPRENbase/) mentioned in this paper are: nucleosome assembly protein 1-like 1 (21327708), prickle-like 1 (23308518), phosphorylase kinase β (4505783), FLJ32421/BROFTI (55665085), RasD2/Rhes (4092830), RhoH (4757770), Rab28 long isoform (4758994), RhoQ (51460469), EH domain binding protein 1 (45501001), DnaJ-homolog A4 (21758015), 72kDa inositol polyphosphate-5-phosphatase E (38327539), WD+tetratricopeptide repeats protein 1 (41018470), G gamma 1 (2695592), G gamma 2 (14589849), rhodopsin kinase (4506529), H-Ras (4885425), R-Ras (5454028), and RhoB (37718739).

Accession numbers (GI numbers) from GenBank (http://www.ncbi.nlm.nih.gov/Genbank) of proteins mentioned in this paper are: ankyrin-repeat containing proteins from canarypox (40555979) and fowlpox virus (41023315), Mimivirus Rab-like protein (55819093), and Mimivirus DnaJ-like molecular chaperone (55819138).

Additional accession numbers of clusters from HumanPRENbase (http://mendel.imp.ac.at/sat/PrePS/HumanPRENbase/) can be found in Tables 2 and 3.

## Abbreviations

FT: farnesyltransferase
FTI: FT inhibitor
GGT1: geranylgeranyltransferase type I
GGT2: geranylgeranyltransferase type II
MCL: Markov chain clustering
NCBI: National Center for Biotechnology Information
NR: nonredundant protein sequence database
pF: protein prenylated by FT but not GGT1
PF: prenyltransferases
pFGG: protein prenylated by FT and GGT1
pGG: protein prenylated by GGT1
PrePS: Prenylation Prediction Suite
REP: Rab escort protein
TLC: thin layer chromatography
